# Pathology is a critical aspect of preclinical aging studies

**DOI:** 10.3402/pba.v3i0.22451

**Published:** 2013-08-20

**Authors:** Warren Ladiges, Yuji Ikeno, Denny Liggitt, Piper M. Treuting

**Affiliations:** 1 Department of Comparative Medicine, School of Medicine, University of Washington, Seattle, WA, USA; 2 Department of Pathology, Barshop Institute for Longevity and Aging Studies, The University of Texas Health Science Center at San Antonio, San Antonio, TX, USA; 3 Geriatric Research, Education and Clinical Center, Audie Murphy VA Hospital, San Antonio, TX, USA

**Keywords:** pathology, aging, mouse, histopathology grading, lifespan, health span

## Abstract

Experimental design for mouse aging studies has historically involved lifespan, but it is now clear that survival data without pathology data limit the information that can be obtained on aging animals. This limitation becomes more serious when interventions of any sort are implemented. Pathology gives an insight into the health of an animal by revealing lesions not readily observable in the live animal. As such, it is a snapshot of disease conditions at the time of death. Therefore, a long-term goal is to establish pathology information as an essential component of studies involving health span and lifespan of aging animals. Given that pathology assessment is essential to help define the progression of lesions associated with aging, the real challenge is including it in aging studies because there is currently a lack of specialized expertise and resources. An increase in the level and scope of pathology assessment of tissues from old mice involved in aging studies is needed. A focus on the correlation of pathology data with longitudinal and cross-sectional lifespan data and health span physiology data can be established by enhancing standard histologic assessment of lesions observed in tissues from old mice. An environment for the development and integration of pathology data into aging studies of mice is needed to encourage more pathologists and other scientists to specialize in pathology of aging, and establish relevant standards to compare with other species including humans. Such results will have an important positive impact on aging studies because of the significant empowerment on data analyses and interpretation.

Pathology is the study of events associated with the gross, histological, and cellular conditions considered abnormal. We are defining pathology as the detection and description of cellular abnormalities in tissues at the end of life (longitudinal) or at specific time points of aging, for example, 18, 25, 32 months (cross-sectional) associated with health span. The assessment can be comprehensive and look at all major organs, or it can be selective and look at certain organs, for example, heart or brain. Generally for studies on lifespan, there is an interest in defining the presence and severity of lesions in as many organs as possible given the constraints for time and resources. Pathology crosses a wide range of disciplines and has an underlying focus of addressing mechanisms using a systematic morphological examination to define the progression of age-associated lesions (1). Pathology assessment can provide detailed observations on the type and severity of lesions present. These can be graded to provide a score and used to correlate with physiology function. Pathology also gives an insight into the health of an animal by revealing lesions not observable in functional assays or with gross observation. Pathology provides the context for molecular, biochemical, and other functional data gathered during the study. Together, these data result in a comprehensive phenotype that can be utilized to study aging pathophysiology and interventions.

It is widely acknowledged that pathology information is an essential component of studies involving aging animals (2–4). Not only one must know the survival rate of the colony of animals that is used in aging studies, but it is also critical that the lesions associated with senescence be characterized. Much of the early research in gerontology suffered greatly from the lack of well-documented pathology data. It is now clear that survival data without pathology data limit the information that can be obtained on aging animals (5). This limitation becomes critical when interventions of any sort are studied as it is difficult to conclude from survival data alone if changes in lifespan arise because aging has been altered by the experimental manipulation, that is, whether a broad spectrum of disease processes have been modified, which is predicted if aging has been altered, as opposed to only one or two disease processes. The best examples are studies using calorie restriction (CR), since CR is considered to be the most effective intervention of aging. The anti-aging effects of CR are associated with reduced incidence and/or delayed occurrence of age-related pathologies. For example, the histology findings of C57BL/6 mice demonstrated that CR suppresses the incidence of both neoplastic and non-neoplastic lesions compared to *ad libitum* fed mice (6). In addition, CR suppresses the severity of various diseases, which indicates that the progression of diseases is delayed by CR. Furthermore, the total number of histopathology lesions, which dramatically increases with age, is suppressed by CR in C57BL/6 mice.

Pathology assessment of aging cohorts has a number of advantages that can enhance other aging endpoints as it validates lifespan extension data by defining lesion burden and health status of the individual or cohort at death. For example, comprehensive histopathology provides information on the types and severity of lesions present making it possible to correlate the contributing causes of morbidity or death with different cohorts and physiology phenotypes.

However, assessment and interpretation of histologic lesions in tissues from old animals requires a highly trained person with appropriate experience. Unfortunately, at present there are a limited number of pathologists trained in assessing lesions in tissues from aging rodents, making it challenging to conduct a pathology assessment.

## Correlation of pathology data with lifespan and health span

There is a need to establish standard procedures for adding pathology to aging studies and to provide a means of interpreting lesions seen in tissues of old mice in a consistent manner (7, 8). As an example, histopathology data were generated from cross-sectional cohorts of 24- and 32-month-old mice in a health span study at the University of Washington. Severity scores were assigned for specific lesions in certain organs such as the lungs, heart, skeletal muscle, and kidney for each mouse in the two age cohorts. These scores are currently being correlated with functional data from each respective mouse to determine if there is any correlation with physiology phenotype, such as heart function determined by echocardiography, skeletal muscle performance determined by running ability and grip strength or pulmonary and renal function tests, observed when the animal was still alive (manuscript submitted for publication). The data can be used in two ways. First, it validates the functional assessment procedure and the in-life data generated indicating a perturbation in physiology performance. Second, it provides an insight and context into diseases present in a given individual mouse and cohort.

In addition to collecting data on the majority of lesions, it is possible to conduct a global assessment of the degree of comorbidity in each mouse. The disease burden, which is defined as the total number of significant lesions in an animal (9–11), provides an index of the total body accumulation of tissue injury. Disease burden increases more than twofold with age in C57BL/6 mice, and anti-aging manipulations such as dietary restriction and mitochondrial targeted anti-oxidant expression have been shown to reduce disease burden in end of life pathology studies (9, 10).

## Integration of pathology into the aging study design

Design of aging studies is influenced by a number of factors depending on the aims and objectives of the project (5, 12, 13). The most demanding requirements are for longitudinal lifespan studies involving either the effect of a genetic modification or the effect of a therapeutic compound. If these studies are designed properly, there should be adequate numbers of mice at the end of life for pathology assessment and determining statistical significance. Some aging projects aim to study physiology function and pathology concurrently. This type of study would test mice on a longitudinal survival basis over several age time points. In order to do pathology at each age time point, separate cohorts of mice would have to be included, a longitudinal functional testing cohort and a pathology cohort terminated at each time point. The possibility of conducting an aging study such as this is greatly increased with the availability of aged mice from the National Institute on Aging, on an age-, strain-, and sex matched-basis.

Another factor to be considered in integrating pathology into aging studies is mouse strain. It is well known that the number of old mice required to obtain a statistically valid number of lesions is strain dependent (5). For example, lung cancer is much more common in old BALB/c mice compared to C57BL/6 mice, whereas old C57BL/6 mice have a much greater incidence of lymphoma (7). Aging studies focusing on either of these conditions would need fewer animals of the respective background. Four-way or eight-way cross mice represent a more heterogeneous genetic background and in general do not have concentrated spikes in commonly occurring age-related lesions so larger numbers of animals would be needed to establish statistical significance.

## Necropsy observations and collection and processing of tissues

Standard protocols on necropsy, collection, and fixation of tissues for comprehensive histology examination in mice have been published (14, 15) and can be modified to fit study needs in keeping with specific goals of aging research and pathologists’ preferences for tissue handling. A general comprehensive necropsy protocol to ensure the proper and consistent collection of tissues in aging studies is the critical first step in obtaining high-quality pathology data (16). Ideally, necropsy is performed on all mice including those found dead. However, histology data may be limited from those carcasses due to autolysis. After euthanasia using approved methods, necropsy should be conducted systematically to ensure that all tissues are examined and collected according to study design, necropsy data sheets with tissue collection checklists and tables for body and organ weights are useful. Digital photography of the dissected carcass is strongly recommended to augment the necropsy records in the pathology database especially if laboratory staff or students and not the study pathologist perform necropsy. Fixation of tissues is most often achieved via immersion in 10% neutral buffered formalin. Other fixatives may be used according to study design. Fixed tissues are trimmed using a standard protocol (17–19).

Pathology assessments are usually performed at the end of a study and after sizable resources have already been invested into mouse housing, physiology, and lifespan assessments. Unfortunately, all too often the comprehensive and systematic analysis of disease phenotypes is reduced or eliminated. This results in loss of critical data or in erroneous interpretation (20, 21). An increase in the quality and scope of pathology assessment of tissues is needed during the initial planning phases of aging research proposals. In addition, the paraffin-embedded and frozen blocks associated with comprehensive histology are an important archival resource for the development of new morphological and molecular investigations (22). These samples can provide opportunities for investigators to conduct morphologic and molecular assessments (e.g. immunohistochemistry, *in situ* hybridization) of aging and age-related pathology in various tissues leading to new avenues of investigation. These samples could also be utilized as a resource for tissue microarray, other morphological analyses, or DNA microarray analyses combined with micro-dissection techniques using laser capture microscopy. Archival pathology samples may also serve as study materials to aid in establishing consensus criteria for lesion characterization, classification, and severity scoring between pathologists working on aging rodent models.

## Summary

Aging is characterized by progressive declines in the function of multiple organ systems and progressive increases in many neoplastic and chronic degenerative non-neoplastic diseases. Aging studies are lengthy, labor intensive, and resource intensive, so it is critical to maximize data from these types of investments. Experimental design for mouse aging studies has historically involved lifespan, but lifespan is only one endpoint. Other endpoints include health span assessment, including physiologic function and pathology. Physiology assessment, such as for cognition, cardiac function, and muscle strength, is now being recognized with increasing interest in aging studies and is relatively easy to integrate into study designs depending on the procedure. Pathology as an aging endpoint crosses a wide range of disciplines and has an underlying focus of addressing mechanisms using a pathology basis to define the progression of age-associated lesions. Pathology can provide detailed observations on the type and severity of lesions present. An environment for the development and integration of pathology data into aging studies of mice will encourage more pathologists and other scientists to specialize in pathology of aging, and establish relevant standards to compare with other species including humans. Such results would be expected to have an important positive impact on aging studies because of the significant empowerment on data analyses and interpretation.
